# Modeling the amplification of epidemic spread by individuals exposed to misinformation on social media

**DOI:** 10.1038/s44260-025-00038-y

**Published:** 2025-04-02

**Authors:** Matthew R. DeVerna, Francesco Pierri, Yong-Yeol Ahn, Santo Fortunato, Alessandro Flammini, Filippo Menczer

**Affiliations:** 1 Luddy School of Informatics, Computing, and Engineering, Indiana University, Bloomington, IN USA; 2 Department of Electronics, Information and Bioengineering, Politecnico di Milano, Milano, Italy

**Keywords:** Epidemiology, Interdisciplinary studies, Communication

## Abstract

Understanding how misinformation affects the spread of disease is crucial for public health, especially given recent research indicating that misinformation can increase vaccine hesitancy and discourage vaccine uptake. However, it is difficult to investigate the interaction between misinformation and epidemic outcomes due to the dearth of data-informed holistic epidemic models. Here, we employ an epidemic model that incorporates a large, mobility-informed physical contact network as well as the distribution of misinformed individuals across counties derived from social media data. The model allows us to simulate various scenarios to understand how epidemic spreading can be affected by misinformation spreading through one particular social media platform. Using this model, we compare a worst-case scenario, in which individuals become misinformed after a single exposure to low-credibility content, to a best-case scenario where the population is highly resilient to misinformation. We estimate the additional portion of the U.S. population that would become infected over the course of the COVID-19 epidemic in the worst-case scenario. This work can provide policymakers with insights about the potential harms of exposure to online vaccine misinformation.

## Introduction

Social factors, such as information sharing, play a crucial role in shaping the dynamics and epidemiology of infectious diseases^1,2^. For instance, a population’s willingness to adopt public health measures (or lack thereof) largely determines their successes or failures^3,4^. A population’s behavioral response to outbreaks can be influenced by mass media, as witnessed during the 2009 H1N1 influenza pandemic^5^, or by social media and the anti-vaccination movement^6–9^.

**Fig. 1:**
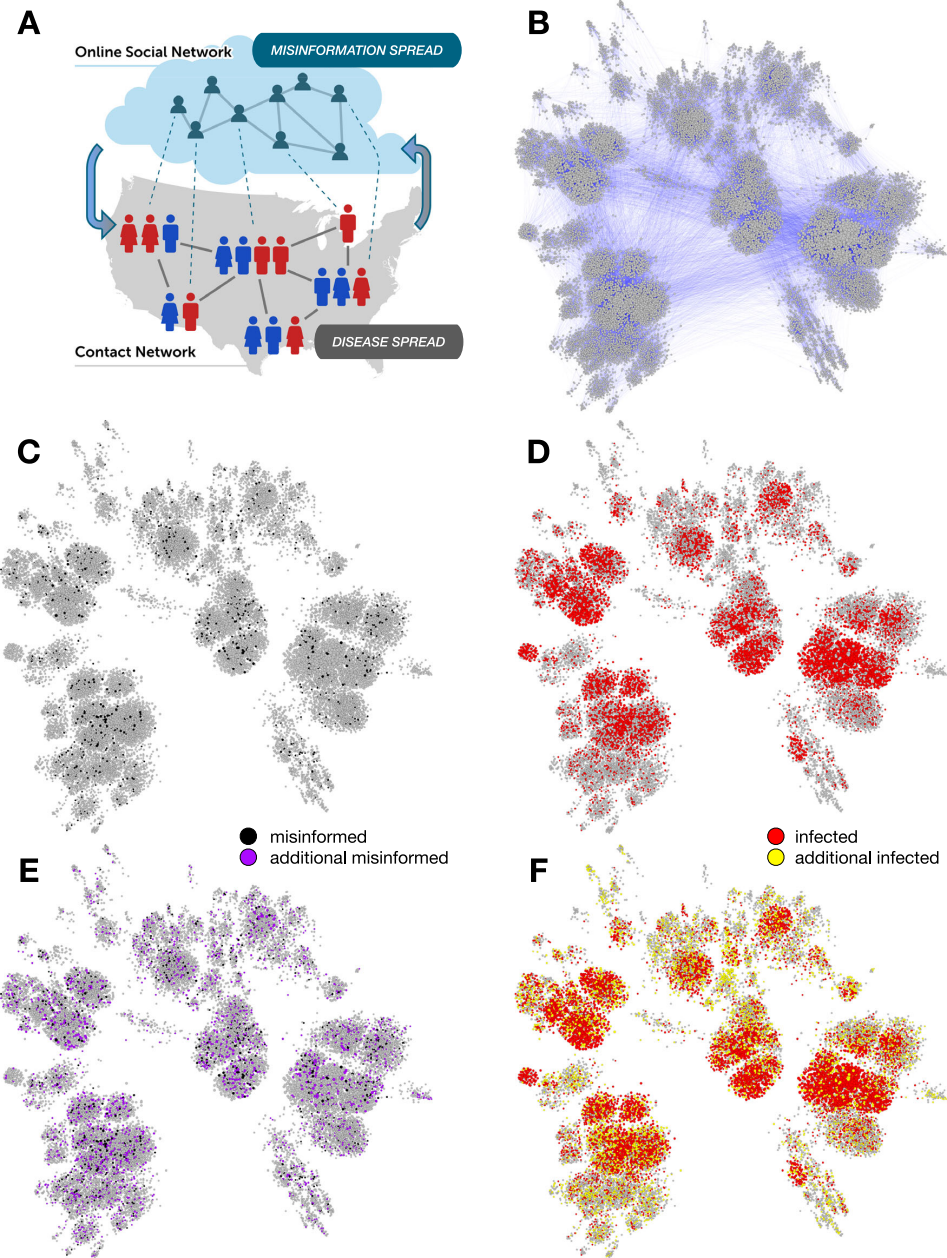
The spread of misinformation affects the transmission of disease. **A** Schematic illustration of the misinformation and contact networks. Online social networks foster misinformation dissemination while physical contact networks, such as those that connect co-workers in an office or pupils in a school, facilitate disease transmission. Dotted links indicate that the same people participate in both networks, which have different topologies; e.g., the information network tends to have stronger political homophily while the contact network tends to have stronger geographic homophily. We focus on the impact of misinformation spread on disease transmission (downward arrow), while the opposite effect (upward arrow, e.g., individuals ceasing to share misinformation due to illness) falls outside the scope of this investigation. **B** A contact network based on 0.01% county population samples. Nodes are sized based on degree (number of contacts). In a scenario with limited spread of misinformation (black nodes in **C**), the simulations of disease spread lead to a number of infected individuals (red nodes in **D**). In a scenario where the misinformation spreads more widely (purple nodes in **E**), more individuals get infected (yellow nodes in **F**).

A great deal of work has explored how to model the influence of human behavior on the spread of infectious diseases^10,11^. Here we focus on risky behaviors affecting disease transmission that are associated with misinformed individuals. Misinformation spreading on social networks has been linked to poor compliance with COVID-19 public health guidance^12^. Greater exposure to unreliable news articles about COVID-19 vaccines has been linked to an increase in vaccine hesitancy and a decrease in vaccination rates at both state and county levels in the United States^13,14^. Exposure to online misinformation has also been shown to increase vaccine hesitancy in laboratory experiments^15^. This is particularly detrimental during vaccination campaigns as clusters of individuals adopting anti-vaccination opinions can make it challenging for a population to reach herd immunity^16,17^. Proper management of epidemic crises in the modern age thus requires the understanding of the complex relationship between the spread of (mis)information through online social networks and the spread of disease through physical contact networks (Fig. 1).

Agent-based simulations have shown that misinformation may impede the suppression of epidemics in various ways^18–21^. One model estimated that between March and November 2021, misinformation caused at least 198 thousand additional COVID-19 cases, 2800 additional deaths, and $299M in additional hospital costs in Canada^22^. However, there is a growing need to strengthen the connections between simulation results and real-world outcomes by integrating real-world data from social media^23,24^.

We address this challenge by proposing a data-informed epidemic model that takes both the distribution of misinformed individuals and a physical mobility network into account. Using this data, we augment the susceptible infected recovered (SIR) model to account for a subpopulation of “misinformed” individuals. We refer to this as the susceptible misinformed infected recovered (SMIR) model. We explore how the misinformed group can affect the larger, ordinary population using a multi-level agent-based simulation based on two large, data-informed networks: a social network where misinformation spreads and a contact network where the disease can propagate. A contact network of approximately 20 million nodes is constructed by leveraging large-scale Twitter data, county-level voting records, and cell phone mobility data. We incorporate theoretically extreme values of the parameter responsible for the epidemic transmission to evaluate best- and worst-case scenarios about the impact of misinformed individuals on the spread of disease and obtain quantitative bounds on the harm caused by misinformation. The proposed model lets us move beyond simplified experimental settings to assess the impacts of misinformation^25^.

## Results

**Fig. 2:**
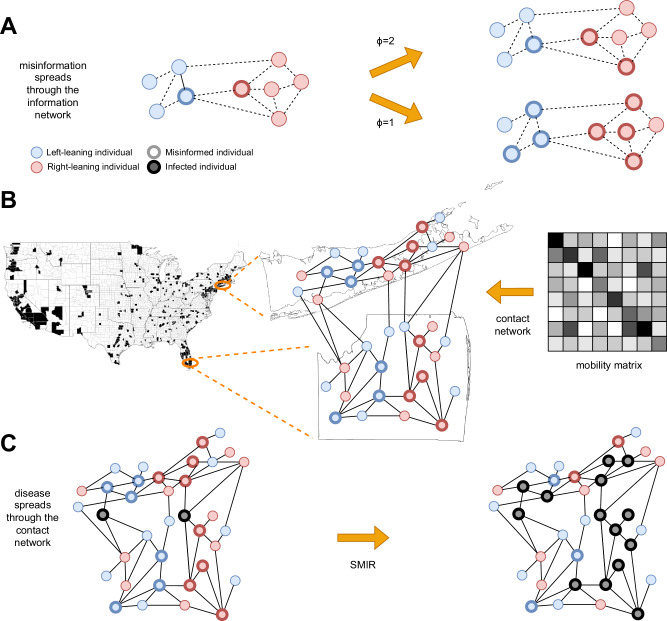
An idealized example of our multi-level modeling framework. **A** Spread of misinformation through an information network (dashed lines). Colors represent ideological homophily. Nodes with bold borders are misinformed about the epidemic. The misinformation spreads through a complex contagion (linear threshold) model; two scenarios show that a lower threshold *ϕ* leads to more misinformed nodes. **B** Construction of the contact network (solid lines) for counties with sufficient information diffusion data (in black) to provide reasonable estimates about the fraction of misinformed individuals. Note that these counties account for 63.52% of U.S. voters. Each location’s population size and ideological mix are based on empirical data, and misinformed individuals are based on the information diffusion model. Links among individuals within and between locations are based on empirical mobility data. **C** The infection spreads through the contact network (black nodes), according to the SMIR model.

We utilize a multi-level, agent-based model to examine the influence of misinformation on epidemic spread. Our approach combines an empirically derived information network with a contact network calibrated with real-world data, as illustrated in Fig. 2. Information diffusion is modeled by leveraging a large set of users of a popular social media platform. Epidemic simulations are subsequently conducted on contact networks populated with misinformed individuals.

We start from a large collection of English-language discussions taking place on Twitter about COVID-19 vaccines^26^. From ~9 months of this data (January 4–September 30, 2021), we geolocate over 2 million U.S. users who shared almost 26 million tweets and focus on accounts in 341 U.S. counties containing more than 200 Twitter users each. We also infer an account’s political alignment and whether they shared any likely misinformation (see the “Methods” section). Twitter is not representative of the U.S. population, and people also access information in other ways, such as traditional media and word of mouth. However, this social media platform serves as one large, realistic network through which people share information about the disease.

With this data, we build a directed and weighted information diffusion network, in which an edge (*i* → *j*, *w*) indicates that *j* retweeted *i* *w* times. There are various ways to model the infodemic^27^. We simulate the spread of misinformation on this network, as illustrated in Fig. 2A. Accounts that share or reshare posts containing misinformation are considered misinformed. These accounts serve as the initial *seeds* from which misinformation proliferates, with exposure to this content likely concentrated within the wider network^28^. Many users may not actively participate in content sharing; for instance, only about half of U.S. Twitter users engage in sharing^29^. Even without active sharing, exposure to misinformation or misleading content can still influence individual behavior^6,15^.

To account for users who may be misinformed through exposure, we employ a single-step linear threshold opinion-spreading process^30^. While many social influence models have been proposed^31^, this is a simple way to capture complex contagion, according to which individuals may require multiple exposures to misinformation before they become misinformed themselves^32–34^. Let a linear threshold *ϕ* represent the minimum number of misinformed friends needed for an ordinary node to become misinformed. If the total number of misinformed friends of *i* is greater than or equal to *ϕ*, *i* is marked as misinformed (*M*). The remaining nodes are marked as ordinary susceptibles (*O*). We can interpret *ϕ* as a measure of “resilience” to misinformation; as it increases, individuals require more exposure to misinformation to be converted to the misinformed group. Conversely, we can think of *ϕ* as inversely related to intent or motivation to engage with low-credibility content^35^. Note that since we explore the full range of *ϕ* values, the following results are unaffected whether the threshold is defined based on the number of users or the number of retweets.

**Fig. 3:**
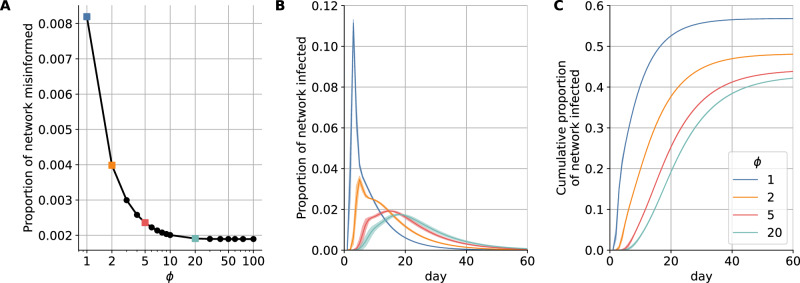
More misinformed individuals lead to a larger portion of the network becoming infected. Decreasing the resilience *ϕ* (**A**) increases the size of the misinformed subpopulation, leading to (**B**) faster infection spreading and (**C**) a greater cumulative number of infections. In panels (**B**, **C**) lines and corresponding shaded regions represent the mean and standard deviation across simulations, respectively.

Figure 3A shows how *ϕ* influences the number of misinformed individuals within the retweet network. With strong resilience (*ϕ* > 10), exposure to misinformation does not have much effect and few nodes are converted to the misinformed group. Conversely, when resilience to misinformation is very low (as in the simple contagion case *ϕ* = 1), all nodes exposed to a misinformation-containing post are converted to the misinformed group. Through this process, empirically observed misinformation-sharing behavior leads to information networks with misinformed subpopulations of varying sizes based on different *ϕ* values.

We generate contact networks for different thresholds (1 ≤ *ϕ* ≤ 20) to compare the impact of misinformed subpopulations of different sizes. Given a threshold *ϕ* and the corresponding information network, we aim to construct a physical contact network containing empirically calibrated misinformed subpopulations (Fig. 2B). The process begins by selecting a sample of individuals from each county within the information network. As party affiliation has been identified as a risk factor associated with excess mortality during the COVID-19 pandemic^36^, county samples are constructed to match the percentage of Republicans and Democrats who voted in the 2020 U.S. presidential election. For each county, we add the sampled nodes to the physical network marked as misinformed (*M*) or ordinary susceptible (*O*), based on their label within the retweet network. Sampling with replacement allows us to select individuals such that the overall proportions of Republicans and Democrats match the voting records. A 10% sample leads to *N* ≈ 20 million nodes. A network based on a much smaller sample is illustrated in Fig. 1B. This process captures empirical measurements of the ideological split, relative population size, and quantity of misinformed individuals in each county. It also allows us to account for the known link between the ideological motivations of users and their exposure to misinformation^14,28^. We add contact network edges by leveraging cell phone mobility data that provides the probability of an individual traveling within and between counties. See Methods for details.

Disease-spreading dynamics on the contact network are simulated using the SMIR model (Fig. 2C). As in the standard SIR^37^, a parameter *β* describes the average number of infected individuals generated by an infected individual in a time unit. We can express $\beta =p\bar{k}$ in terms of two critical parameters that affect the spreading dynamics: the density of the contact network, captured by its average degree $\bar{k}$, and the transmission probability *p*. Infected individuals recover with rate *γ*.

We extend this epidemic model to account for misinformed and ordinary subpopulations. Ordinary individuals are considered to be well-informed about public health guidelines, such as social distancing, mask-wearing, and vaccination. Mitigation measures such as social distancing decrease $\bar{k}$, while those such as masking and vaccination decrease *p*. Misinformed individuals, having been exposed to untrustworthy information, are assumed to be less likely to follow these recommended behaviors, thereby increasing the risk of infection for themselves and others^38^. A simple way to model the combined effects of misinformation on these behaviors through a single parameter is to set $\bar{k}=25$, a high value corresponding to the average number of daily contacts prior to the COVID-19 pandemic^39^ and use extreme values of *p* to capture worst- and best-case scenarios. An effective reduction of contacts, resulting, for example, from social distancing or lockdowns, can be captured by decreasing the *p* parameter.

We, therefore, model the refusal of any mitigation measures by selecting the maximum value *p*_M_ = 1 for misinformed individuals. In contrast, we model the adoption of several mitigation measures by selecting an extremely small value *p*_O_ = 0.01 for ordinary individuals. The former scenario portrays a realistic number of interactions during non-pandemic times, accompanied by high transmission rates due to the absence of preventive measures, such as social distancing, mask-wearing, or vaccinations. The latter demonstrates decreased daily interactions and reduced transmission rates resulting from the implementation of these preventive measures. Using the empirically calibrated contact networks in conjunction with these extreme parameters, the simulation approach allows us to bound the best- and worst-case scenarios in a data-informed manner (see the “Methods” section for more information).

The effects of the misinformed subpopulation size on the daily incidence of infection (illustrated in Fig. 1C–F on a small network) are quantified in Fig. 3B on a large network (10% sample). The worst case capturing a heavily misinformed population (*ϕ* = 1) corresponds to an additional 9% of the population being infected at peak time (a six-fold increase) compared to a resilient population following expert guidance in the best-case scenario (*ϕ* = 20). The peak also occurs approximately two weeks earlier. The cumulative effect is also significant, with an additional 14% of the population infected over the course of the epidemic compared to cases with a more resilient population—a 32% relative increase (Fig. 3C).

We explored alternative scenarios for the ratio *p*_M_/*p*_O_ through a mean-field approximation. Predictably, as this ratio gets larger, the infected population increases and the peak infection occurs earlier. We also considered different sample sizes for the empirical network and found that the main results are robust. These analyses can be found in Supplementary Information.

## Discussion

Exposure to online health misinformation is associated with risky behaviors such as vaccine hesitancy and refusal^14^. There is also experimental evidence suggesting a causal link^6,15,40^. While one study found no evidence that misinformation reduces intent to vaccinate, the authors report that they did not have sufficient power to detect small effects^41^. Assuming an association exists between exposure to health misinformation on one particular social media platform and risky behaviors, this work uses large-scale epidemic simulations to further link the behaviors of misinformed individuals to an accelerated spread of disease. Our model is anchored in empirical data^23,24^ to explore potential outcomes.

Agent-based simulations of the SMIR model let us study the epidemic on empirically calibrated contact networks. By comparing a worst-case scenario, in which individuals become misinformed after a single exposure to low-credibility content, to a best-case scenario where the population is highly resilient to misinformation, the model estimates that the peak of the infection is amplified by a factor of six and accelerated by two weeks. This would result in an additional 14% of the population becoming infected—nearly 47 million Americans based on recent U.S. Census data^42^. The corresponding price tag of vaccine misinformation would be over $143B, using estimated healthcare costs associated with COVID-19 in the U.S.^43^.

While these figures are based on extreme scenarios, they represent an alarming bound on the harm of exposure to online vaccine misinformation. They should provide public health authorities as well as social media platforms with heightened motivation to curb vaccine misinformation, despite the difficulties posed by social media design^44^.

Our results do not address the differential effects of the epidemic on the two populations of ordinary and misinformed individuals. We carry out such an analysis using a mean-field approximation of the model, which assumes all individuals have an equal chance of interacting. The mean-field model demonstrates how the risky behaviors of misinformed individuals can adversely impact those following public health guidelines, worsening outcomes for the entire population (see Supplementary Information). Additionally, we use the mean-field model to explore the role of homophily in the population, i.e., scenarios where misinformed individuals are more likely to be connected to other misinformed individuals and similarly for the ordinary population. We find that increasing homophily can benefit the overall population by protecting ordinary citizens; however, it may also lead to higher infection rates within the misinformed subpopulation (see Supplementary Information).

We acknowledge several limitations in our approach. The model assumes the existence of a causal link between exposure to online misinformation and the adoption of risky behaviors. There is a need for models that can provide support for this assumption beyond existing lab experiments^6,15^.

Using empirical retweet data as a proxy for social connections may not capture potential passive exposure to misinformation. While follower relationships could diminish this limitation, our choice allows us to focus on users who are more likely to be impacted due to their active engagement.

We model a single wave of infection with somewhat arbitrary extreme-case parameters (*p*_O_ = 0.01, *p*_M_ = 1). A broader range of values is explored in a mean-field scenario, along with the effect of the size of the misinformed population (see Supplementary Information). Of course, as *p*_O_ → 0, only the misinformed population can get infected in the model. However, since the mean-field scenario ignores the network structure, its results cannot be directly compared to those of the agent-based model. COVID-19 saw multiple waves of infection with different variants, varying reproduction numbers, levels of immunity, and so on. Future work should attempt to quantify the potential effects of misinformation in more realistic scenarios, where the key parameters *p*_M_ and *p*_O_ could be calibrated on empirical surveillance data from particular regions and time periods.

We also assume uniform resilience to misinformation for all individuals during the information diffusion process, although this attribute likely differs across individuals. Future directions could involve more sophisticated models to account for these heterogeneities. For instance, cognitive models of misinformation acceptance^45^ could be incorporated into the simulation with misinformation exposure data collected from social media. Such integration would enable the transition of individuals from ordinary to misinformed susceptible states throughout the simulation, allowing for a simultaneous examination of opinion and disease dynamics. Some theoretical models have already explored similar approaches and obtained results that align with our findings^18,19^.

Finally, although individual beliefs and behaviors may vary over time, our model simplifies the scenario by dichotomizing individuals into misinformed and ordinary subpopulations and assuming constant transmission rates. Future extensions of the model could account for a feedback loop whereby witnessing local infections could drive changes in behaviors equivalent to the transition of individuals out of the misinformed population^46^.

## Methods

### Twitter and derived data

Twitter posts in the CoVaxxy dataset^26^ were collected in real-time via the stream/filter endpoint of the Twitter Application Programming Interface (API). To capture the online discourse surrounding COVID-19 vaccines in English, a comprehensive set of English-language keywords was carefully curated. Beginning with the initial seeds of “covid” and “vaccine,” a snowball sampling technique^47^ was used to identify co-occurring relevant keywords in December 2020^26^. The resulting list contained almost 80 keywords, available online^48^. To confirm the relevance of the collected tweets to the topic of vaccines, we examined the coverage obtained by incrementally adding keywords, starting with the most common ones. Over 90% of the tweets in 2021 contained at least one of the three most common keywords: “vaccine,” “vaccination,” or “vaccinate.” To infer the location of accounts, we used the Carmen Python library^49^ that leverages self-reported location metadata within user profiles (embedded in tweets). As an account’s location may change over time (captured across multiple tweets), we utilize the most recent location. We geolocate 2,047,800 users residing in all 50 U.S. states, who shared a total of 25,806,856 tweets by mapping self-reported locations to U.S. counties. The information network is constructed from accounts in 341 counties that contain more than 200 Twitter users each. Political alignment is estimated using a third-party list of annotated news sources^50,51^. It is averaged across all the sources shared by each account. Nodes with an estimated alignment greater (smaller) than zero are considered Republican (Democrat). We infer the political alignment of some additional accounts, who did not share links to news sources, using a label-propagation algorithm^52^ on the retweet network. If all of a node’s neighbors have political alignment scores, its score is estimated using the weighted average of its neighbors, with weights based on retweets. The process is iterated until each node without a score has at least one neighbor without a score. Misinformation is defined at the source level. Tweets containing links to articles from a list of low-credibility sources compiled by NewsGuard (score below 60) are labeled as spreading misinformation. This approach is common practice and has been validated in the literature^14,53–56^.

### Contact network edges

To construct edges in our contact network, we utilize SafeGraph cell-phone mobility data^57^, which contains information on the number of people residing in over 200K Census-Block-Groups (CBG) who visited 4.3M Points-of-Interest (POI) in the United States. This data has been widely employed to study human mobility patterns during the COVID pandemic. We used the average daily number of individuals moving during 2019 as a reference for business-as-usual mobility and aggregated all CBGs and POIs at the county level. This aggregation results in a county-by-county matrix *L*, where each element *L*_*xy*_ represents the average daily number of individuals in county *x* moving to county *y* or vice versa. We then normalized *L*_*xy*_ to obtain the average probability of individuals in counties *x* and *y* coming into contact and multiplied by the total number of edges to obtain the expected number of connections between individuals in counties *x* and *y*: ${E}_{xy}=\frac{{L}_{xy}}{{\sum }_{{x}^{{\prime} },{y}^{{\prime} }}{L}_{{x}^{{\prime} }{y}^{{\prime} }}}\frac{\bar{k}N}{2}$ where the sum is over all county pairs and $\frac{\bar{k}N}{2}$ is the total number of edges. Next, we create a physical contact network with *N* nodes by following a procedure akin to a stochastic block model^58^ used to generate networks with localized communities. For each pair of distinct locations *x* and *y*, we draw *E*_*xy*_ edges between random pairs of nodes in *x* and *y*. Additionally, we draw *E*_*xx*_ edges among random pairs of individuals within the same location *x*, representing homogeneous mixing within each county. At the end of the process, the network has the target average degree $\bar{k}$. We use $\bar{k}=25$ and show how this parameter affects the infections in Supplementary Information.

### Simulation details

Agent-based SMIR simulations are initiated by randomly selecting 100 misinformed nodes and designating them as infected. The disease-spreading dynamics are then simulated for 100 steps, which correspond to days. To align with COVID-19 dynamics, we utilize the CDC’s recommended quarantine period of 5 days as our recovery period^59^ (*γ* = 0.2). Each simulation is repeated ten times, and the average outcome is reported.

## Supplementary information

Supplementary Information

## Data Availability

Data are available in a public repository: https://github.com/osome-iu/bounding-misinfo-impact-on-disease-spread.

## Code availability

Code is available in a public repository: https://github.com/osome-iu/bounding-misinfo-impact-on-disease-spread.
