# Supplementary material for: Modeling the amplification of epidemic spread by individuals exposed to misinformation on social media
Source: Npj Complex. 2025 Apr 2;2(1):11. doi: 10.1038/s44260-025-00038-y (PMC11964913; doi:10.1038/s44260-025-00038-y)
Supplement: Supplementary file 1 — Supplementary Information [file 44260_2025_38_MOESM1_ESM.pdf]

Supplementary information

1 Mean-field SMIR model

For both the ordinary and misinformed subpopulations, the Susceptible Misinformed Infected Recovered (SMIR) model replicates the standard SIR compartments, denoted as $S_O/I_O/R_O$ and $S_M/I_M/R_M$, respectively. SMIR adopts distinct transmission parameters for the misinformed (β_M) and ordinary (β_O) groups. (In the agent-based model, these are proportional to p_M and p_O, respectively.) The mean-field approximation assumes that the population is well mixed, ignoring the empirical network structure, and that infected individuals from either group ($I_O + I_M$) can potentially infect *anyone* in the susceptible populations. The mean-field model is governed by the following system of equations:

$$\begin{cases} \frac{dS_O}{dt} = -\beta_O S_O(I_O + I_M), \quad \frac{dI_O}{dt} = \beta_O S_O(I_O + I_M) - \gamma I_O, \quad \frac{dR_O}{dt} = \gamma I_O \\ \frac{dS_M}{dt} = -\beta_M S_M(I_O + I_M), \quad \frac{dI_M}{dt} = \beta_M S_M(I_O + I_M) - \gamma I_M, \quad \frac{dR_M}{dt} = \gamma I_M. \end{cases} \tag{1}$$

To model homophily, we modify the term $I_O + I_M$ in Eq. 1 to account for increased (decreased) contacts within (across) groups, according to the parameter $\alpha \geq 0.5$. When homophily does not play a role ($\alpha = 0.5$), there is an equal probability of interacting with either subpopulation's infected group. We thus obtain:

$$\begin{cases} \frac{dS_O}{dt} = -2\beta_O S_O(I_O\alpha + I_M(1-\alpha)), \quad \frac{dI_O}{dt} = 2\beta_O S_O(I_O\alpha + I_M(1-\alpha)) - \gamma I_O, \quad \frac{dR_O}{dt} = \gamma I_O \\ \frac{dS_M}{dt} = -2\beta_M S_M(I_O(1-\alpha) + I_M\alpha), \quad \frac{dI_M}{dt} = 2\beta_M S_M(I_O(1-\alpha) + I_M\alpha) - \gamma I_M, \quad \frac{dR_M}{dt} = \gamma I_M. \end{cases} \tag{2}$$

Let us denote the proportions of misinformed and ordinary individuals as μ and $1-\mu$, respectively. A proportion $\epsilon = 0.001$ of the population is initially infected, split evenly between the ordinary and misinformed groups. Thus, during the initial state, we have initial values for each compartment: $R_O = R_M = 0$, $S_O = \mu - \frac{\epsilon}{2}$, $S_M = 1 - \mu - \frac{\epsilon}{2}$, $I_M = \frac{\epsilon}{2}$, and $I_O = \frac{\epsilon}{2}$.

To identify a suitable base value for the transmission rate among ordinary susceptibles, we begin by exploring the scenario with no misinformed individuals ($\mu = 1$), setting $\gamma = 0.2$ and varying the transmission parameter in the range $0.02 \leq \beta_O \leq 1$. As is typical of SIR dynamics, Fig. S1 shows that lower β_O values delay and lower the infection peak — the so-called "flattening of the curve." Lower β_O also decreases the total proportion of the population that becomes infected at any point during the epidemic, while higher β_O values increase this proportion. These dynamics are tied to the basic reproduction number $R_0 = \beta/\gamma$: the disease spreading dynamics only reach epidemic levels when $R_0 > 1$, such that an infected individual infects more than one other person on average. This happens when $\beta_O > 0.2$. As R_0 increases, the infection spreads more quickly, the peak infection day occurs sooner, and the proportion of the population that is ultimately infected increases.

We now explore the effect of the recovery rate, again in the scenario with no misinformation or homophily, by setting $\beta_O = 0.3$ and varying the recovery period $\tau = 1/\gamma$ between 1 and 20 days. Fig. S2 shows that when $\tau < 4$, $R_0 < 1$ and the disease does not reach epidemic proportions. At this level, the epidemic takes

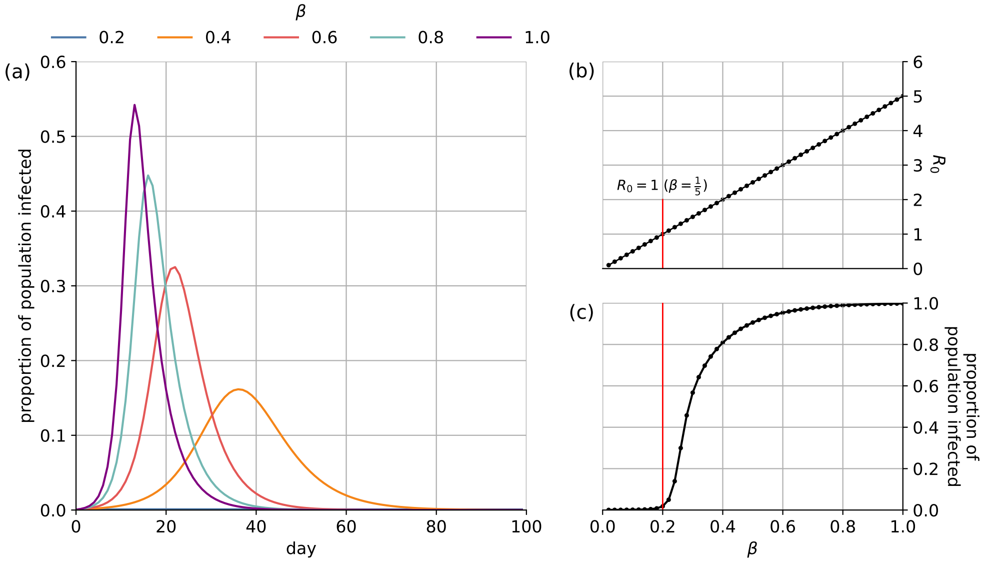

Figure S1: Reducing the transmission parameter β_O decreases the severity of the epidemic. We plot (a) the proportion of the population infected each day, (b) R_0 values for the ordinary population, and (c) the total proportion of the population infected as β_O varies. In (a), the curve for $\beta_O = 0.2$ is difficult to see because the proportion of the population infected remains very low throughout the simulation. Here we do not consider the role of misinformation or homophily.

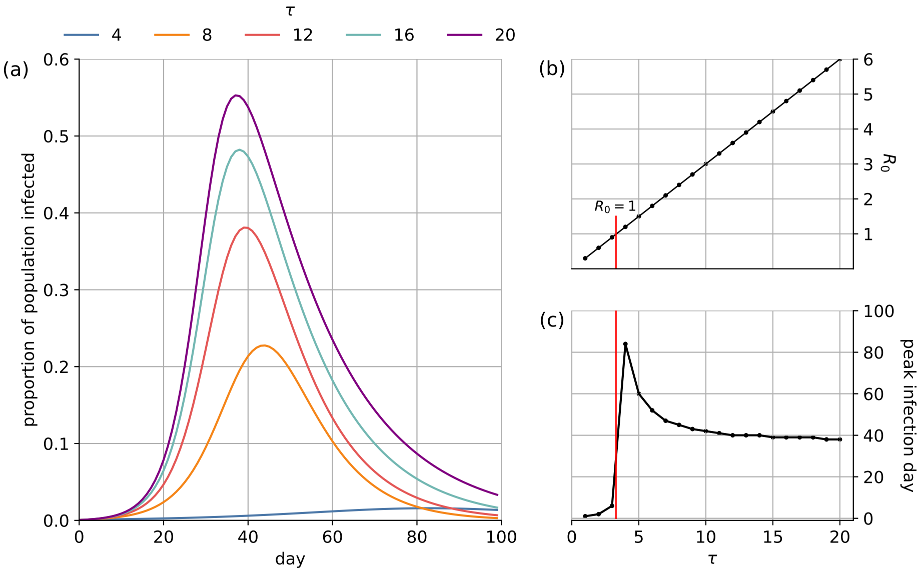

Figure S2: Effects of varying the recovery rate. We plot (a) the proportion of the population infected each day, (b) R_0 values for the ordinary population, and (c) the total proportion of the population infected as a function of the number of days to recover, τ. Here we do not consider the role of misinformation or homophily.

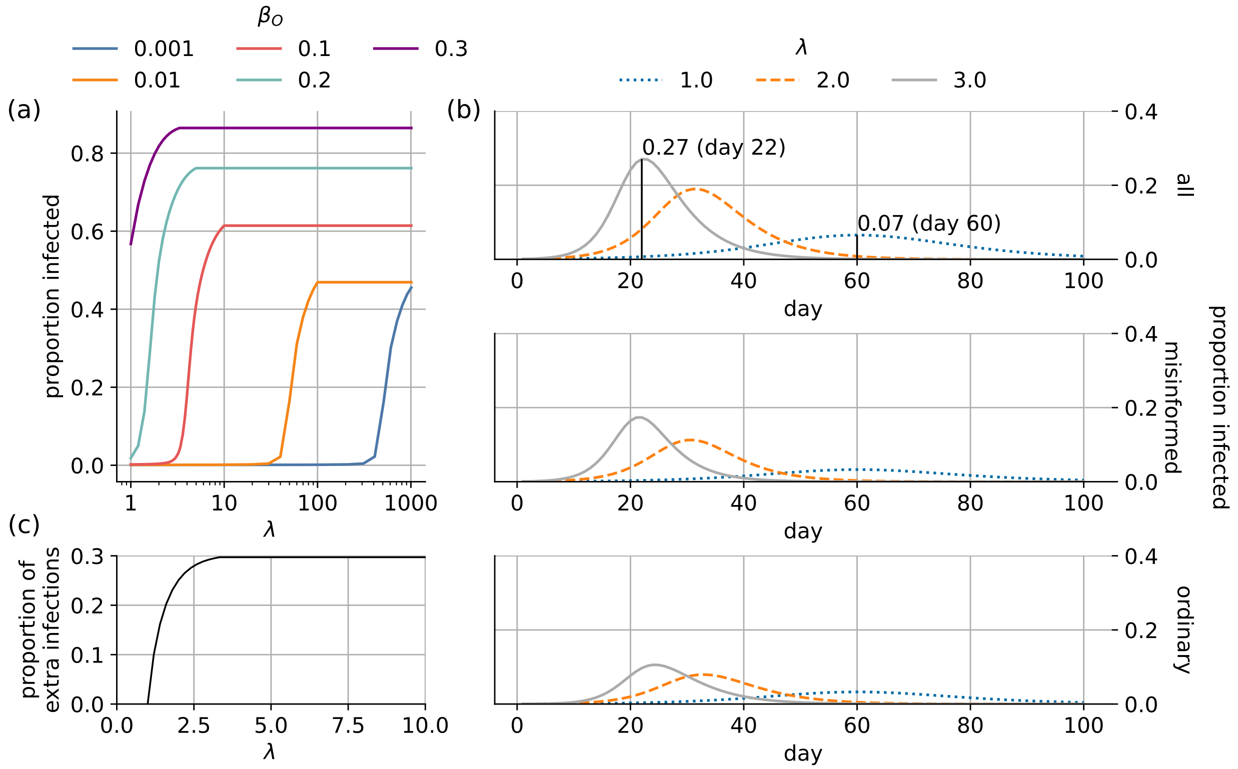

Figure S3: Increasing $\lambda = \beta_M/\beta_O$ accelerates and amplifies the infection. We use $\gamma = 0.2$, and $\mu = 0.5$. (a) Overall proportion of the population infected as a function of λ, for different values of β_O. (b) Proportion of the population infected on each day, for different values of λ using $\beta_O = 0.3$. (c) Extra proportion of the total population that is infected as a function of λ ($\beta_O = 0.3$).

a long time to reach its peak (≈80 days). Increasing τ means that individuals remain infected longer, so the population gets infected faster and the peak infection is reached more rapidly.

In summary, the effects of varying the transmission and recovery parameters are predictable: lower β and higher γ "flatten the curve" and reduce the negative outcomes of an infection. Based on these explorations we set $\tau = 5$ ($\gamma = 0.2$) to align with quarantine recommendations from the CDC[16]. We further set $\beta_O = 0.3$ such that the basic reproduction number is $R_0 \geq \beta_O/\gamma = 1.5$ to ensure epidemic spread within the ordinary population.

2 Mean-field analyses

To explore the effects of risky behaviors by misinformed individuals, let us assume two equally-sized subpopulations ($\mu = 1/2$) and introduce the scaling factor $\lambda = \beta_M/\beta_O \geq 1$. Fig. S3(a) illustrates the increasing negative impact of the misinformed subpopulation on the disease-spreading dynamics as λ becomes larger. If the ordinary population has very low β_O, λ has to be very high for the misinformed population to have an

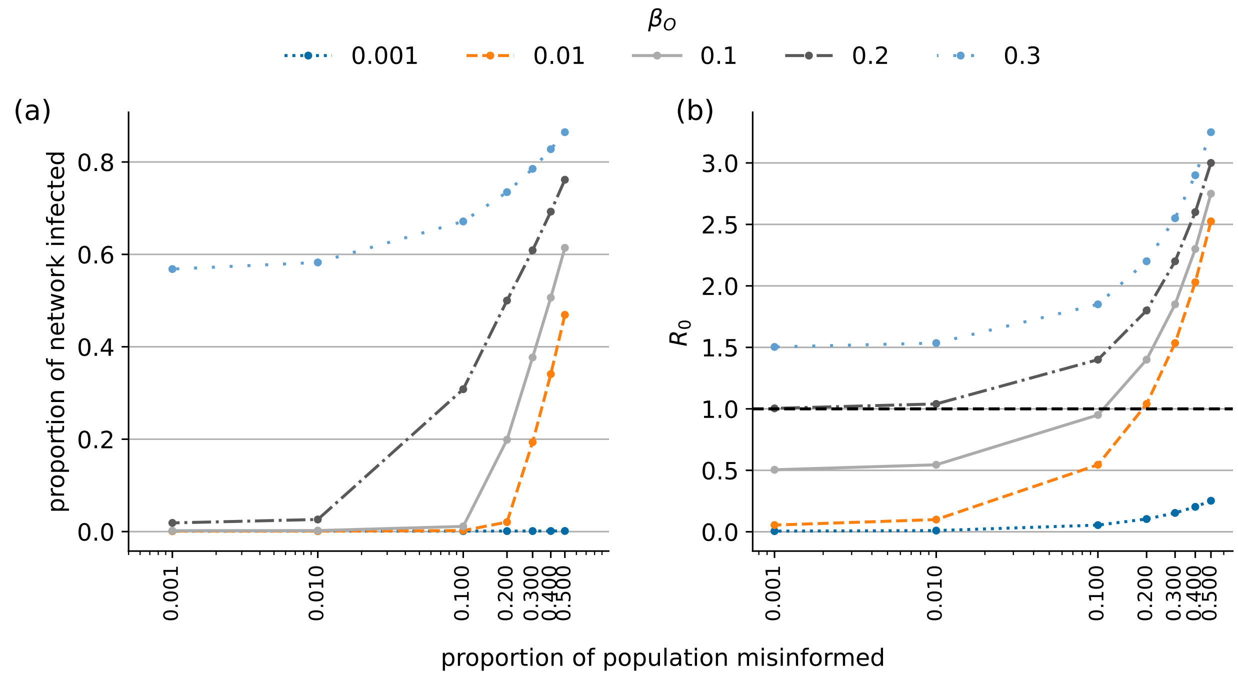

Figure S4: Increasing the initial proportion μ of the population that is misinformed, as well as β_O, increases (a) the size of the network that becomes infected and (b) the average R_0 across the population. Here, we fix $\lambda = 100$ to match the ratio used in the main text.

effect. On the other hand, if β_O is large enough, increasing λ leads to a ceiling effect, as β_M cannot exceed one. The social cost associated with the more risky behaviors by the misinformed group is passed on to the whole network. For example, when $\lambda = 3.0$, peak infection for the entire population is reached 38 days earlier than in the $\lambda = 1$ case (day 22 vs. 60; Fig. S3(b)), leading to an additional 29.3% of the population becoming infected (Fig. S3(c)).

We further explore how the initial size μ of the misinformed population affects the total proportion of the network that ultimately gets infected. We consider various values of β_M and β_O such as to capture the same $\lambda = \beta_M/\beta_O = p_M/p_O = 100$ as in the main text. When μ and β_O are both low, the misinformed population has no impact on the infection (Fig. S4(a)), as $R_0 < 1$ (Fig. S4(b)). However, increasing either parameter crosses the epidemic threshold ($R_0 > 1$) so that a significant portion of the population gets infected.

3 Effect of homophily

Let us explore the effect of homophily among the ordinary and misinformed subpopulation networks. Homophily means that infected individuals are more likely to interact with (and infect) susceptibles from the same subpopulation (ordinary or misinformed) than the other group. The degree of homophily is modeled by a parameter α. When $\alpha = 0.5$, individuals are equally likely to interact within and across groups (no

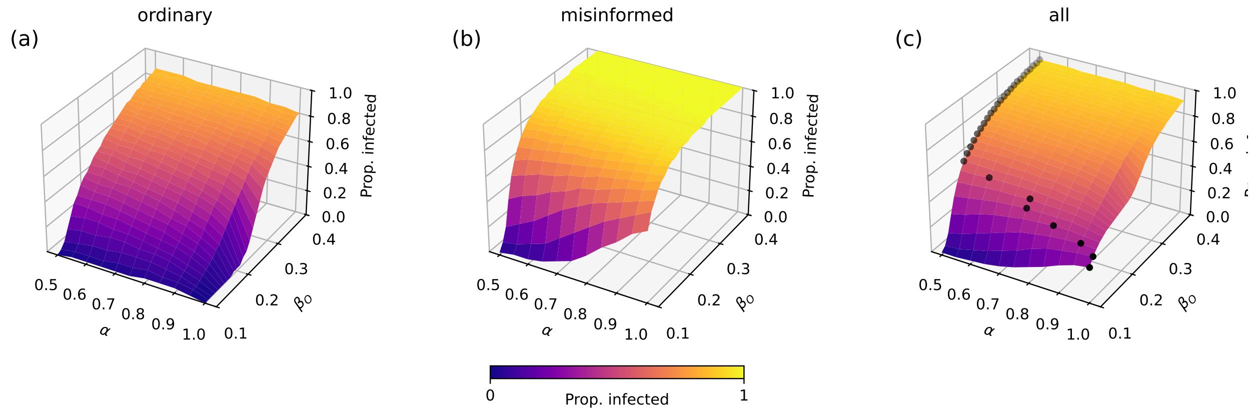

Figure S5: Homophily in the contact network worsens the infection among misinformed individuals, especially for lower transmission rates. The combined effects of transmission and homophily parameters, β_O and α, are examined with the mean-field approximation when $\lambda = 3$, $\gamma = 0.2$, and $\mu = 0.5$. We plot the proportions of infected individuals in (a) the ordinary population, (b) the misinformed population, and (c) the overall population. The maximum proportion of the overall population infected for each β_O is marked with a black dot. When the transmission rate is sufficiently high, homophily benefits the entire population but harms the misinformed group.

homophily), whereas $\alpha = 1$ is the case when homophily is strongest and the subpopulations do not interact with each other (see Methods for details).

Fig. S5 illustrates the effects of homophily ($0.5 \leq \alpha \leq 1$) for different levels of ordinary transmission ($0.1 \leq \beta_O \leq 0.4$). For less infectious disease (low β_O), increasing homophily significantly harms the misinformed group (Fig. S5(b)): the infection remains confined within this group. There is no discernible effect on the ordinary population as long as the two groups interact; when they do not ($\alpha = 1$), we observe a sharp reduction in infections (Fig. S5(a)). For $0.12 < \beta_O < 0.16$, peak infection scenarios coincide with intermediate homophily levels, as indicated by the black dots in Fig. S5(c)[39]. Under these conditions, while increased homophily decreases infections in the general populace, it significantly worsens outcomes for the misinformed group (compare Fig. S5B and C). As β_O increases further, while nearly the entire misinformed population becomes infected regardless of α (Fig. S5(b)), high homophily shields the full population (Fig. S5(c)): ordinary individuals have a lower risk of becoming infected through interactions with misinformed individuals. In summary, homophily offers greater protection to the ordinary group by isolating misinformed communities, which suffer a greater disease burden, exacerbating health disparities[49,53].

4 Robustness analyses

To test the robustness of the main results (Fig. 3C) with respect to the sample size used to construct the contact network, all simulations were rerun after generating contact networks based on the different sampling

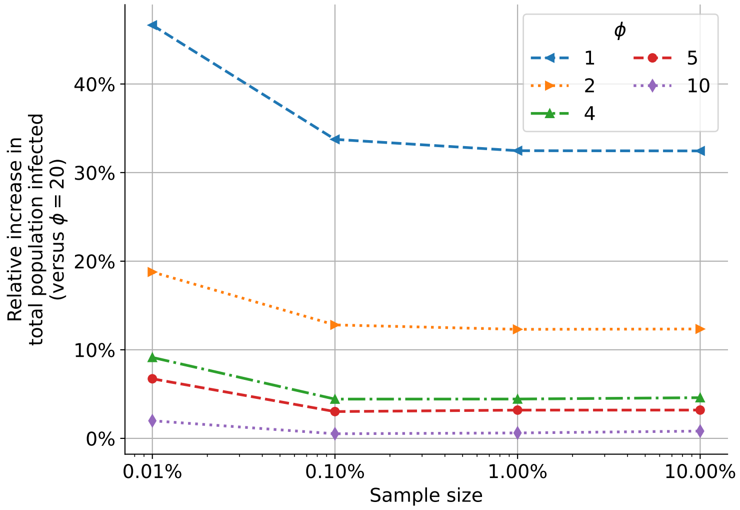

Figure S6: Relative increase in the mean total population infected as a function of the sampling size utilized in the contact network creation process. The $\phi = 20$ scenario, in which the fewest nodes in the network are misinformed, is utilized as the baseline.

percentages between 0.01% and 10%. Fig. S6 shows the relative increase in the percentage of the population that becomes infected as a function of the linear threshold ϕ, using the best-case scenario in which the fewest nodes in the network are misinformed as the baseline. We observe a substantial decrease in the effect of misinformation as the sampling size grows to 1%. However, sample sizes above 1% return nearly identical results. We conclude that using a sample size of 10% (as reported in the main text) is sufficient to rule out any size-induced bias.

Fig. S7 illustrates the impact of the contact network density ($\bar{k}$) on infection dynamics, for $5 \leq \bar{k} \leq 25$. We consider this range because $\bar{k} = 25$ represents pre-pandemic daily social contacts while $\bar{k} = 5$ represents COVID-19 lockdown conditions[34]. As expected, Fig. S7(a) demonstrates that higher $\bar{k}$ leads to increased infections through the population, since the higher contact density provides more opportunities for transmission. But while a larger percentage of the overall population is infected, the relative effect of misinformed individuals decreases. This is because, at higher $\bar{k}$ values, the infected population is already substantial even in the low-misinformation ($\phi = 20$) baseline. The combined effect of these two opposing trends, as shown in Fig. S7(b), is that the additional percentage of infected individuals relative to the $\phi = 20$ scenario reaches a maximum for some intermediate $\bar{k}$. Fig. S7(b) also shows that, consistent with our primary findings, increasing ϕ (misinformed resilience) decreases the infected population. In our main analysis we focus on $\bar{k} = 25$ and model an effective reduction of contacts by decreasing the p parameter.

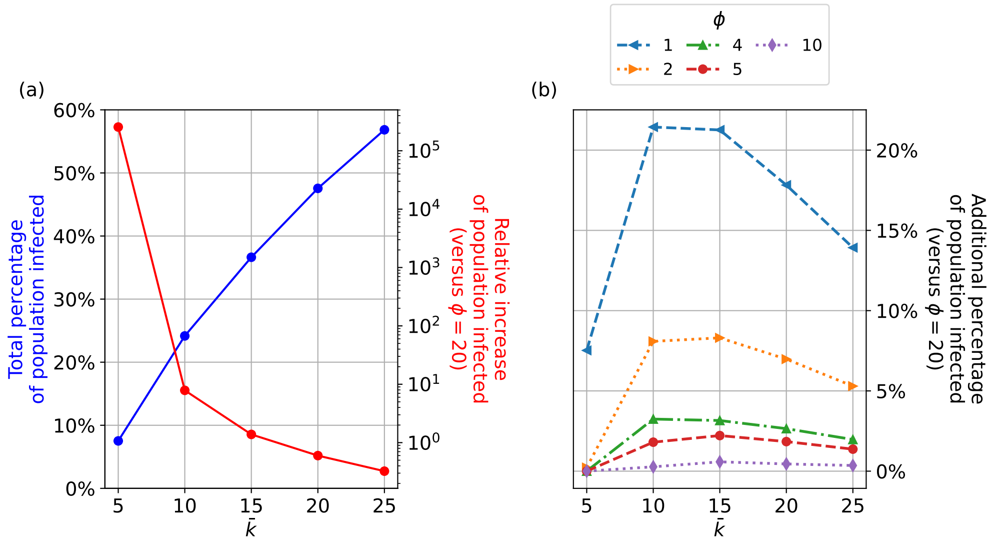

Figure S7: Effects of average contact network degree $\bar{k}$ on infection dynamics. (a) Infected individuals ($\phi = 1$) as a percentage of the overall population and relative to the baseline condition $\phi = 20$, in which the fewest nodes in the network are misinformed. (b) Additional percentages of infected population relative to the baseline condition $\phi = 20$.
